# EspP, an Extracellular Serine Protease from Enterohemorrhagic *E*. *coli*, Reduces Coagulation Factor Activities, Reduces Clot Strength, and Promotes Clot Lysis

**DOI:** 10.1371/journal.pone.0149830

**Published:** 2016-03-02

**Authors:** Kevin H. M. Kuo, Shekeb Khan, Margaret L. Rand, Hira S. Mian, Elena Brnjac, Linda E. Sandercock, Indira Akula, Jean-Philippe Julien, Emil F. Pai, Alden E. Chesney

**Affiliations:** 1 Division of Medical Oncology and Hematology, University Health Network, Toronto, ON, Canada; 2 Department of Medical Biophysics, University of Toronto, Toronto, ON, Canada; 3 Campbell Family Cancer Research Institute, Ontario Cancer Institute, University Health Network, Toronto, ON, Canada; 4 Division of Hematology, Hospital for Sick Children, Toronto, ON, Canada; 5 Department of Laboratory Medicine and Pathobiology, University of Toronto, Toronto, ON, Canada; 6 Department of Biochemistry, University of Toronto, Toronto, ON, Canada; 7 Department of Medicine, University of Toronto, Toronto, ON, Canada; 8 Department of Clinical Pathology, Sunnybrook Health Sciences Centre, Toronto, ON, Canada; 9 Program in Molecular Structure and Function, The Hospital for Sick Children Research Institute, Toronto, ON, Canada; 10 Department of Immunology, University of Toronto, Toronto, ON, Canada; 11 Department of Molecular Genetics, University of Toronto, Toronto, ON, Canada; Stanford University, UNITED STATES

## Abstract

**Background:**

EspP (*E*. *coli* secreted serine protease, large plasmid encoded) is an extracellular serine protease produced by enterohemorrhagic *E*. *coli* (EHEC) O157:H7, a causative agent of diarrhea-associated Hemolytic Uremic Syndrome (D+HUS). The mechanism by which EHEC induces D+HUS has not been fully elucidated.

**Objectives:**

We investigated the effects of EspP on clot formation and lysis in human blood.

**Methods:**

Human whole blood and plasma were incubated with EspP^WT^ at various concentrations and sampled at various time points. Thrombin time (TT), prothrombin time (PT), and activated partial thromboplastin time (aPTT), coagulation factor activities, and thrombelastgraphy (TEG) were measured.

**Results and Conclusions:**

Human whole blood or plasma incubated with EspP^WT^ was found to have prolonged PT, aPTT, and TT. Furthermore, human whole blood or plasma incubated with EspP^WT^ had reduced activities of coagulation factors V, VII, VIII, and XII, as well as prothrombin. EspP did not alter the activities of coagulation factors IX, X, or XI. When analyzed by whole blood TEG, EspP decreased the maximum amplitude of the clot, and increased the clot lysis. Our results indicate that EspP alters hemostasis *in vitro* by decreasing the activities of coagulation factors V, VII, VIII, and XII, and of prothrombin, by reducing the clot strength and accelerating fibrinolysis, and provide further evidence of a functional role for this protease in the virulence of EHEC and the development of D+HUS.

## Introduction

Enterohemorrhagic *Escherichia coli* (EHEC), especially those of serogroup O157:H7, are known causative agents of hemorrhagic colitis (HC) and diarrhea-associated hemolytic-uremic syndrome (D+HUS). While Shiga toxins are thought to be the primary virulence factor in HC and D+HUS [1], other virulence factors have also been identified, including cytolethal distending toxin (CDT)-V, EHEC hemolysin (EHEC-hly), and subtilase cytotoxin [2].

*E*. *coli* secreted serine protease, large plasmid encoded, (EspP) was first identified in 1997 as a secreted protein encoded by the large plasmid (pO157) of *E*. *coli* O157:H7 [3]. To date, EspP has been found in a further 55 of 106 Shiga toxin-producing *E*. *coli* serotypes as five subtypes: α, β, γ, δ, and ε [4,5]. Of these, EspPα is associated with highly pathogenic serogroups (O157, O26, O111, and O145) [4,6]. EspP is a member of the serine protease autotransporters of *Enterobacteriaceae* (SPATE) and is among the most abundant secreted proteins of EHEC [7]. Like other SPATEs, EspP consists of an N-terminal “passenger domain” and a C-terminal “β domain”. The β domain targets the protein to the bacterial outer membrane and facilitates translocation of the passenger domain across that membrane. Following its translocation onto the bacterial cell surface, the passenger domain is separated from the β domain by an autoproteolytic cleavage reaction within the barrel pore that is mediated by the β domain, and released free into the extracellular milieu [8,9]. The catalytic proteolytic function of the protein resides within the N-terminal globular domain [10]. The EspP passenger domain contains an extended right-handed parallel β-helical stalk domain preceded by an N-terminal globular domain. The functional significance of the β-helical stalk domain remains unclear.

In their initial characterization of this protease, Brunder *et al*. showed EspP to cleave human coagulation factor V (FV). FV is a 330 kDa single-chain glycoprotein present in human plasma [11] and a critical component of coagulation [12]. The authors then hypothesized that local degradation of FV by EspP, secreted by EHEC attached to the gastrointestinal mucosa, may result in a reduction of FV activity leading to prolongation of coagulation time; EspP may therefore play a role in the pathophysiology of D+HUS by increasing haemorrhage into the gastrointestinal tract [3].

In the present study, we examined the effects of EspP on coagulation parameters, clot formation, and clot lysis in human plasma and whole blood in an *in vitro* setting and found that EspP alters hemostasis *in vitro*.

## Materials and Methods

### Ethics

The study was approved by the Sunnybrook Health Sciences Centre Research Ethics Board. Written informed consent was provided by participants prior to enrolment in the study. All clinical investigations were conducted according to the principles expressed in the Declaration of Helsinki.

### Protein production and whole blood collection

Wild type EspP (EspP^WT^) as well as the S236A mutant form of this protease (EspP^S263A^), which has abrogated serine protease activity, were purified as previously described [10], adjusted to 10 mg/mL in phosphate-buffered saline containing 25% (v/v) glycerol (PBS-G), and stored at -70°C until use. Whole blood (500 mL) was collected into citrate phosphate double dextrose (CP2D) anticoagulant, following informed consent, by a phlebotomist *via* venipuncture at the antecubital fossa using a 16-gauge needle from each of 6 randomly selected hereditary hemochromatosis (HH) patients undergoing regular therapeutic phlebotomies. There is currently no established relationship between HH and thrombosis or coagulopathy that would be expected to alter the effects of EspP on coagulation parameters, clot formation, or clot lysis.

### Incubation of human whole blood and human plasma with EspP

Immediately following blood collection, 900 μL of EspP^WT^ (10 mg/mL in PBS-G) was added to 8.1 mL of citrated whole blood, mixed by gentle inversion, separated into three 3-mL aliquots and incubated at 37°C with occasional gentle mixing. Incubations with each of buffer (PBS-G) alone, BSA (10 mg/mL in PBS-G), and EspP^S263A^ (10 mg/mL in PBS-G) served as negative controls. At 0.5, 2, and 4 h, one 3-mL aliquot from each set was removed from the incubation chamber and 0.34 mL from each aliquot was immediately assayed for its clotting behavior by thrombelastography (see below). The remaining 2.66 mL from each aliquot were centrifuged (10 min, 4°C) at 1,000 × g to recover the plasma fraction, which was flash-frozen on dry ice, and stored at -70°C. At a later date, each aliquot was thawed for 5 min in a 37°C water bath and assayed for coagulation factor activities. The entire procedure was repeated with blood from one donor at a fixed incubation time of 4 h but with the EspP^WT^ concentration varied at 0.1 mg/mL, 0.5 mg/mL, and 1.0 mg/mL.

To determine whether the effects of EspP on coagulation are dependent on the presence of cellular components (platelets, red blood cells, and white blood cells) present in whole blood, the above procedure was repeated with the following modifications: 40 mL of fresh citrated whole blood was first centrifuged (10 min, 25°C) at 1,000 x g to recover the plasma fraction. 300 μL of EspP^WT^ (10 mg/mL in PBS-G) were then added to 2.7 mL of the recovered plasma, separated into three 1-mL aliquots, and incubated at 37°C with occasional agitation. At 0.5, 2, and 4 h, one 1-mL aliquot from each set was removed, flash-frozen on dry ice, and stored at -70°C. At a later date, this plasma fraction was thawed for 5 min at 37°C and assayed for coagulation factor activities.

### Thrombelastography

Thrombelastographic assays of whole blood were performed using a Computerized Thrombelastograph Hemostasis Analyzer (TEG) Model 5000 (Haemoscope Corp, Niles, IL). 0.34 mL of citrated fresh blood reaction mixture was placed into a TEG sample cup, recalcified by addition of 20 μL of 200 mM CaCl_2_, and monitored for 1 h in the TEG analyzer. The following parameters were determined: R (reaction time), the latency between placing the recalcified blood sample into the thrombelastograph and when the first fibrin strands form; K, and α angle, measures of the kinetics of clot formation; MA (maximum amplitude), a measure of the maximum strength of the clot; and LY30, a measure of the percentage of clot lysis 30 min after MA is reached.

### Analysis of coagulation factor activities

1 mL of thawed plasma fraction from each reaction mixture (sample) described above was loaded into an Automated Coagulation Laboratory Total Operational Performance (ACL TOP) hemostasis analyzer (Instrumentation Laboratory, Lexington, MA) and assayed for thrombin time (TT), prothrombin time (PT), and activated partial thromboplastin time (aPTT), as well as for activities of coagulation factors V, VII, VIII, IX, X, XI, XII, and prothrombin. All reagents, calibrators, controls, and diluents used on the ACL TOP were from the HemosIL group of reagents (Instrumentation Laboratory, Lexington, MA), and all assays were performed under routine laboratory conditions in the single test mode using the ACL TOP’s recommended protocols. These protocols are briefly described here.

To measure PT, 50 μL of sample was incubated at 37°C with 100 μL of RecombiPlasTin 2G (a liposomal preparation that contains recombinant human tissue factor relipidated in a synthetic phospholipid blend and combined with calcium chloride, buffer, and a preservative), which acts to initiate coagulation. The optical density of the mixture was then monitored at 671 nm and an endpoint in seconds was obtained based on clot formation.

To measure aPTT, 50 μL of sample was incubated at 37°C with 50 μL of SynthASil APTT reagent (a buffered reagent which contains synthetic phospholipid for optimal platelet-like activity and a highly defined non-settling colloidal silica for optimal activation of the contact phase of coagulation) for 200 s. 50 μL of 20 mM CaCl_2_ was then added to initiate coagulation. The optical density of the mixture was monitored at 671 nm and an endpoint in seconds was obtained based on clot formation.

To measure TT, 80 μL of sample was incubated at 37°C with 80 μL of TT reagent (a buffered solution of bovine albumin, 1.9 UNIH/mL bovine thrombin, and 100 mM CaCl_2_), which acts to initiate coagulation. The optical density of the mixture was then monitored at 671 nm and an endpoint in seconds was obtained based on clot formation.

To measure prothrombin activity, 29 μL of sample was mixed with 87 μL of Factor Diluent at 37°C. To 50 μL of this diluted sample was then added 50 μL of FII deficient plasma and 100 μL of RecombiPlasTin 2G. Coagulation was monitored by observation of the optical density of the mixture at 671 nm and compared with a calibration curve to calculate prothrombin activity.

To measure FV, FVII, and FX activities, 12 μL of sample was mixed with 108 μL of Factor Diluent at 37°C. To 50 μL of this diluted sample was then added 50 μL of FV, FVII, or FX deficient plasma and 100 μL of RecombiPlasTin 2G. Coagulation was monitored by observation of the optical density of the mixture at 671 nm and compared with a calibration curve to calculate FV, FVII, or FX activity; the factor for which activity was measured was the one depleted in the factor deficient plasma used.

To measure FVIII, FIX, FXI, and FXII activities, 10 μL of sample was mixed with 90 μL of Factor Diluent at 37°C. To 25 μL of this diluted sample was added 25 μL of FVIII, FIX, FXI, or FXII deficient plasma and 50 μL of SynthASil APTT Reagent. After incubation at 37°C for a further 200 s 50 μL of 20 mM CaCl_2_ was added to initiate coagulation. The optical density of the mixture was monitored at 671 nm and compared with a calibration curve to calculate FVIII, FIX, FXI, or FXII activity; the factor for which activity was measured was the one depleted in the factor deficient plasma used.

### Determination of lipopolysaccharide (LPS) levels in sample preparations

The levels of LPS in the PBS-G, BSA, EspP^WT^, and EspP^S263A^ samples were determined using the HEK-Blue LPS Detection Kit 2 (InvivoGen, San Diego, CA) and the recommended protocol therein. Briefly, serial dilutions of the LPS standards were prepared in a 96-well round-bottom microplate at a final volume of 20 μL and a final LPS concentration of 1.0, 0.50, 0.25, 0.125, 0.062, 0.031, 0.016, 0.08, 0.004, and 0 EU/mL. In parallel, serial dilutions of PBS-G, BSA (0.1 mg/mL stock), EspP^WT^ (0.1 mg/mL stock), and EspP^S263A^ (0.1 mg/mL stock) were prepared in a 96-well round-bottom microplate at a final volume of 20 μL and a final dilution factor of 1, 1/3, 1/10, 1/30, 1/100, 1/300, 1/1000, 1/3000, 1/10000, and 0. All dilutions were performed using LPS-free water. To each well was then added 160 μL of HEK Blue cells (40,000 cells/mL stock) and the microplate was incubated at 37°C in a CO_2_ (5%) incubator for 18 h. 20 μL of the supernatant from each well was then transferred to a 96-well flat-bottom microplate, supplemented with 180 μL of Quanti-Blue, and incubated at 37°C for 6 h. The absorbance at 650 nm of each well was then measured and compared with a standard curve to calculate LPS levels.

### Cleavage of human coagulation factors V and VIII by EspP

To assay for cleavage of human coagulation factors V and VIII, 0.1 μg of EspP^WT^ was incubated (5–15 h at 37°C) with 4.2 μg of purified human coagulation factor Va (BioPur AG, Budendorf, Switzerland) in 20 mM HEPES buffer with 150 mM NaCl, pH 7.4, or 10 μg of purified recombinant human coagulation factor VIII (Bayer Inc, Toronto, ON) in 50 mM TEA with 150 mM NaCl, pH 7.4, in a total reaction volume of 20 μL. Incubations with buffer alone served as negative controls. Cleavage products were analyzed by SDS-PAGE followed by silver (FVa) or SYPRO Orange (Molecular Probes, Eugene, OR; FVIII) staining.

### Statistical analysis

Descriptive statistics with mean and 95% confidence interval were calculated for all continuous variables. Relationships between incubation time and change in coagulation factor activities, measures of coagulation, and thrombelastographic parameters were explored with paired-samples t-test. Differences in the changes of coagulation factor activities, measures of coagulation, and thrombelastographic parameters between whole blood and plasma, as well as LPS levels were explored by independent-samples t-test. Unless otherwise indicated, all statistical analyses were two-sided tests with 0.05 as the critical level of significance (α level). P values are reported to three decimal places with P values less than 0.001 reported as <0.001. All analyses were computed with SPSS ver. 20 (IBM).

## Results

### EspP^WT^ reduces the activities of specific human coagulation factors

**Fig 1:**
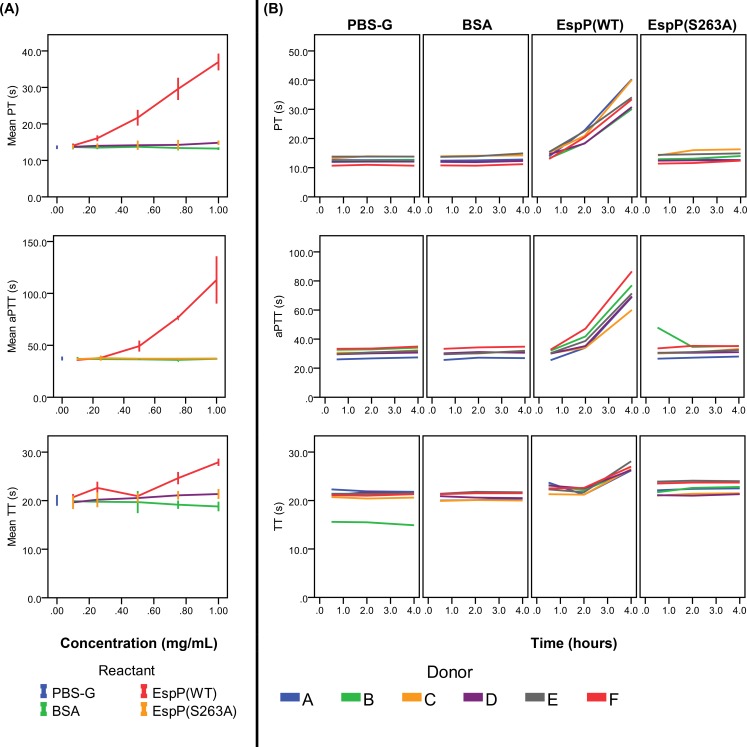
Concentration- and time-dependent prolongation of PT, aPTT and TT by EspP^WT^. (A) Fresh-frozen plasma from donor B was incubated (37°C, 4 h) with buffer alone (PBS-G) or with 0.1, 0.5, or 1.0 mg/mL of BSA, EspP^WT^, or EspP^S263A^. Prothrombin time (PT), activated partial thromboplastin time (aPTT), and thrombin time (TT) were then determined for each sample. Shown are mean ± 95% CI values derived from three parallel experiments. Note that most error bars are smaller than the height of the data point markers due to the small variability between measurements. (B) Fresh-frozen plasma from 6 donors (A to F) was incubated (37°C) with buffer alone (PBS-G) or with 1.0 mg/mL of BSA, EspP^WT^ (WT), or EspP^S263A^ (S263A) for 0.5, 2, or 4 h. Prothrombin time (PT), activated partial thromboplastin time (aPTT), and thrombin time (TT) were then determined for each sample. Shown are values obtained from single measurements.

**Fig 2:**
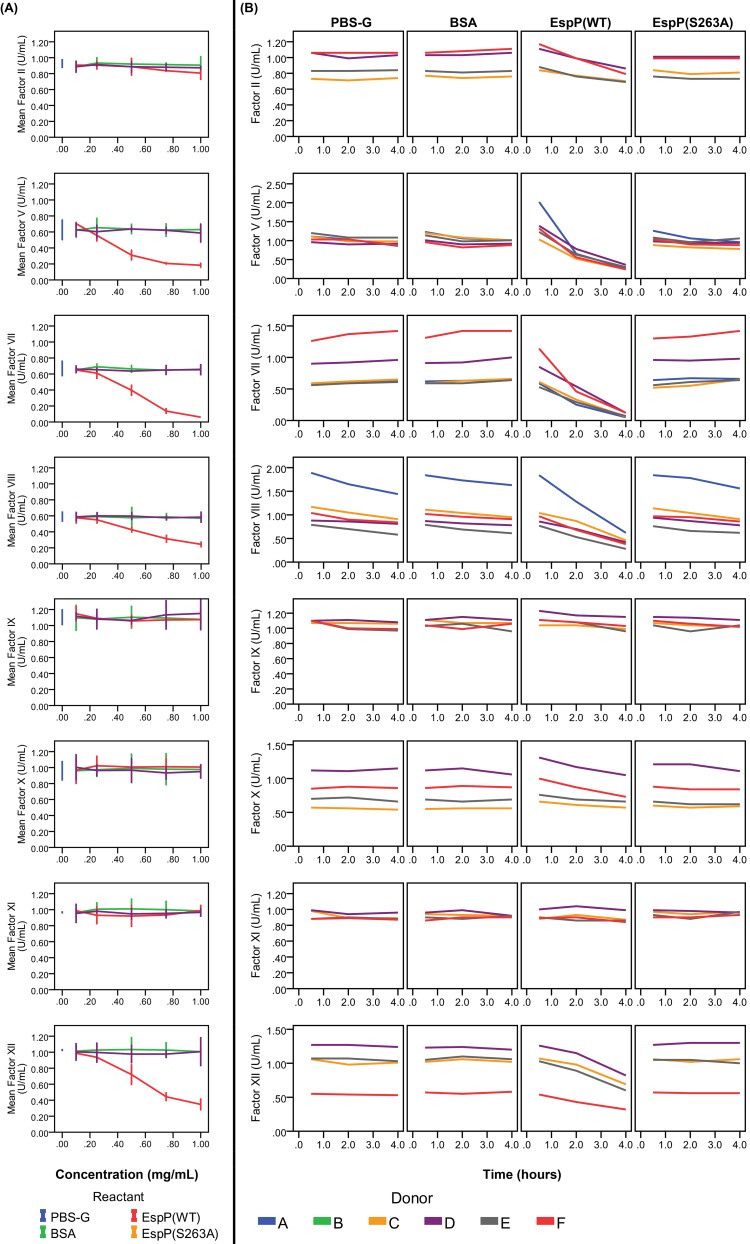
Selective concentration- and time-dependent reduction in coagulation factor activities by EspP^WT^. (A) Fresh-frozen plasma from donor B was incubated (37°C, 4 h) with buffer alone (PBS-G) or with 0.1, 0.5, or 1.0 mg/mL of BSA, EspP^WT^, or EspP^S263A^. Residual activities of coagulations factors II, V, VII, VIII, IX, X, XI, and XII were then determined for each sample. Graphically shown are mean ± 95% CI values derived from three parallel experiments. Note that these error bars are shorter than the height of the data point markers for many of the data points. (B) Fresh-frozen plasma was incubated (37°C) with buffer alone (PBS-G) or with 1.0 mg/mL of BSA, EspP^WT^, or EspP^S263A^ for 0.5, 2, or 4 h. Residual activities of coagulation factors II, V, VII, VIII, IX, X, XI, and XII were then determined for each sample. Shown are values obtained from single measurements. Blood samples from donor A were not analyzed for residual activity of factors II, IX, X, XI, and XII. Blood samples from donor B were not analyzed for any factor activity.

Treatment of whole blood with EspP^WT^ resulted in prolongation of PT, aPTT, and TT in a concentration- and time-dependent manner (Fig 1). Following incubation of whole blood with 1 mg/mL EspP^WT^ for 4 h, PT was prolonged by 32.7 s (P<0.001), aPTT by 77.5 s (P = 0.001) and TT by 10.2 s (P<0.001) relative to incubation with BSA. No significant prolongation in PT, aPTT, or TT was observed in any of the negative controls, including EspP^S263A^, regardless of incubation time or concentration. Furthermore, treatment of whole blood with EspP^WT^ resulted in reduced activities of coagulation factors V, VII, VIII, XII, and prothrombin in a concentration and time-dependent manner (Fig 2). Incubation of whole blood with 1.0 mg/mL EspP^WT^ for 4 h reduced the residual activity of coagulation factor V by 0.66 U/mL (P<0.001), factor VII by 0.73 U/mL (P = 0.004), factor VIII by 0.58 U/mL (P = 0.002), and factor XII by 0.46 U/mL (P = 0.005) relative to incubation with BSA. Although the coagulation activity of prothrombin was also significantly reduced (by 0.17 U/mL, P = 0.005, relative to incubation with BSA), the magnitude of this reduction was modest relative to that observed for coagulation factors V, VII, VIII, and XII. EspP^WT^ did not significantly alter the coagulation activities of factors IX, X, and XI in either whole blood or plasma (Fig 2). Identical magnitudes of prolongation in PT, aPTT, and TT as well as reduction in residual activities of factors V, VII, VIII, XII, and prothrombin were observed when incubations were performed in plasma and in whole blood.

**Fig 3:**
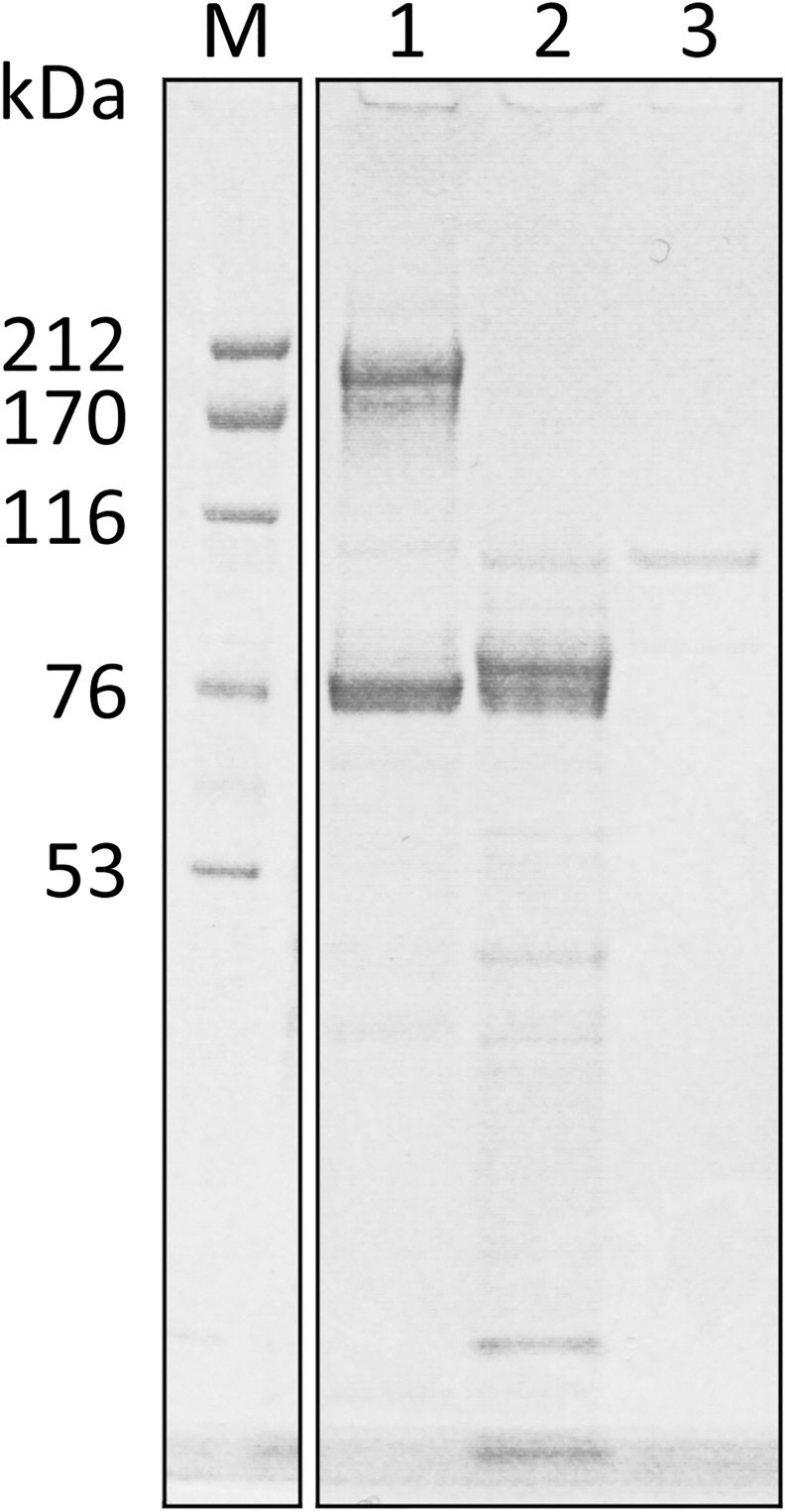
Cleavage of human coagulation factor VIII by EspP. Purified human coagulation factor VIII (10 μg) was incubated for 16 h at 37°C with buffer alone (50 mM TEA, pH 7.4 and 500 mM NaCl; lane 1) or with 0.1 μg EspP^WT^ (lane 2). EspP^WT^ incubated with buffer alone was loaded in lane 3. Molecular weight markers were loaded in lane M. Protein bands were visualized by SYPRO Orange staining.

To confirm that the reduction of factor VIII activity was due to proteolytic cleavage by EspP, purified human coagulation factor VIII was incubated with EspP^WT^ for 16 h at 37°C (Fig 3). FVIII is a highly glycosylated heterodimer composed of a ~200 kDa heavy chain and a ~80 kDa light chain. Incubation of purified human coagulation factor VIII with EspP^WT^ resulted in the disappearance of the band around 200 kDa, corresponding to the heavy chain of the highly glycosylated human factor VIII (hFVIII), and the appearance of a band at around 80 kDa but slightly larger than the light chain of human FVIII, when compared to hFVIII incubated with buffer alone. Further degradation products below ~60 kDa were also observed (Fig 3).

### EspP^WT^ reduces clot strength and promotes clot lysis

**Fig 4:**
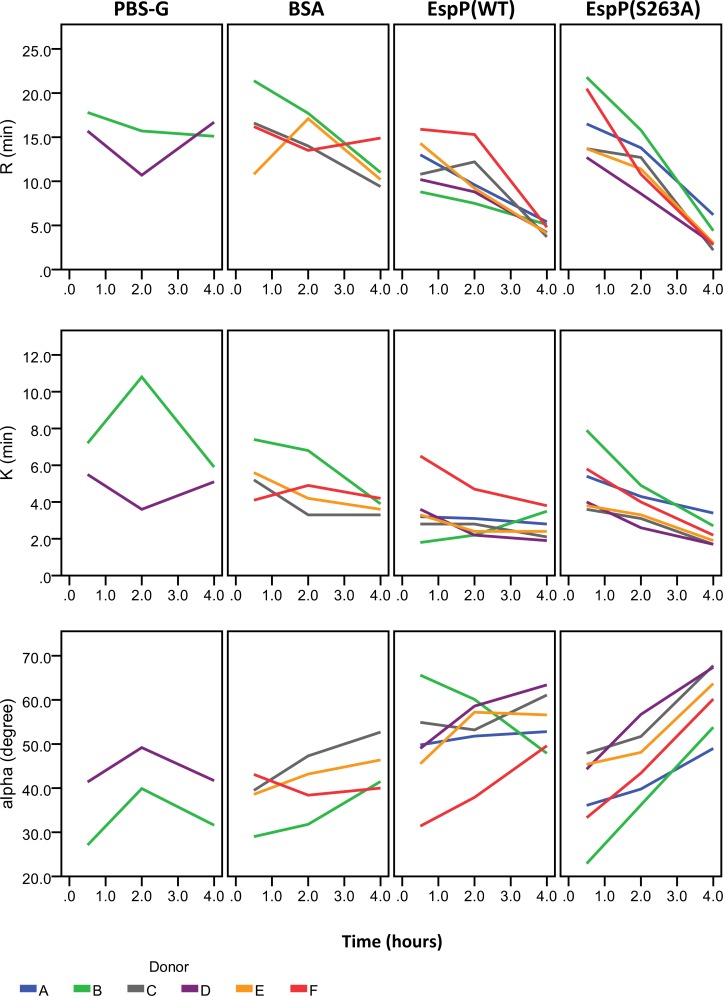
Clot formation kinetics of whole blood treated with EspP. Fresh citrated whole blood from 6 donors (A to F) was incubated with EspP^WT^ (1.0 mg/mL) or with EspP^S263A^ (1.0 mg/mL) for 0.5, 2, or 4 h, then analyzed by TEG. Blood from donors B and D were additionally incubated with buffer alone (PBS-G) as a negative control. Blood from donors B, C, E, and F were additionally incubated with BSA (1.0 mg/mL) as a negative control. Shown are the observed reaction time (R-time), clot formation time (K-time), and α-angle. These results can be explained by LPS contamination.

Analysis by thrombelastography revealed that whole blood treated with EspP^WT^ had a reduced R-time and K-time and an increased α-angle in a manner dependent on treatment time (Fig 4). When whole blood was incubated with 1.0 mg/mL EspP^WT^ for 4 h, R-time reduced by 7.0 min (P = 0.007), K-time reduced by 0.9 min (P = 0.023), and α-angle increased by 9.5° (P<0.001) relative to incubation with BSA. Significant incubation time-dependent reductions in R-time and K-time, and increase in α-angle, were also observed when whole blood was treated with 1 mg/mL EspP^S263A^ (Fig 4). Following incubation of whole blood with 1mg/mL EspP^S263A^ for 4 h, R-time reduced by 8.3 min (P = 0.008), K-time reduced by 1.7 min (P<0.001), and α-angle increased by 17.4° (P<0.001) relative to incubations with BSA. The magnitude of reduction in R-time in EspP^S263A^-treated whole blood was not significantly different from EspP^WT^ at 2 and 4 h (P = 0.390 and 0.070, respectively). The magnitude of reduction in K-time in whole blood by EspP^S263A^ was not significantly different from EspP^WT^ at 0.5, 2, and 4 h (P = 0.132, 0.133, and 0.152, respectively). The magnitude of increase in α-angle was not significantly different between EspP^S263A^- and EspP^WT^-treated whole blood at 0.5 and 2 h (P = 0.104 and 0.144, respectively) and was only marginally significant at 4 h (P = 0.046).

**Fig 5:**
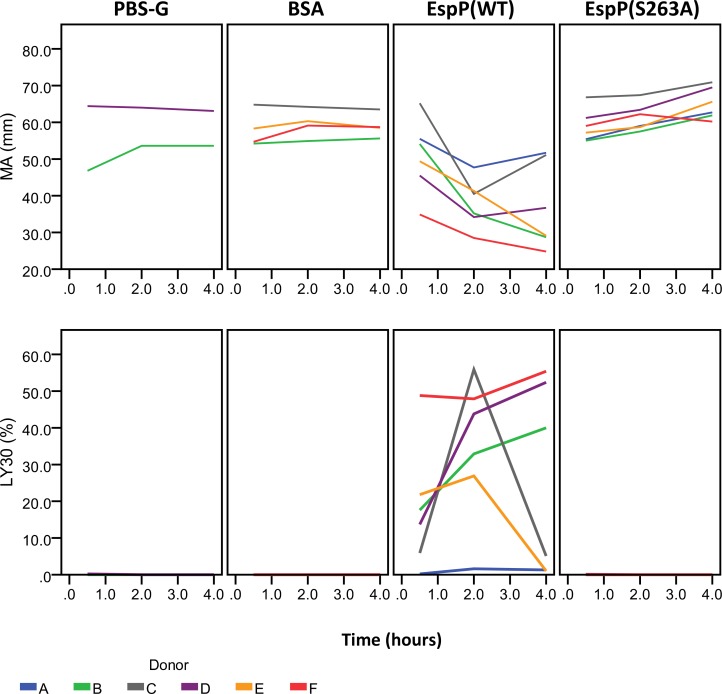
EspP accelerates fibrinolysis in only five of six additional donors. Fresh citrated whole blood from 6 donors (A to F) was incubated with EspP^WT^ (1.0 mg/mL) or with EspP^S263A^ (1.0 mg/mL) for 0.5, 2, or 4 h, then analyzed by TEG. Blood from donors B and D were additionally incubated with buffer alone (PBS-G) as a negative control. Blood from donors B, C, E, and F were additionally incubated with BSA (1.0 mg/mL) as a negative control. Shown are the observed maximum amplitude of clot (MA) and percent clot lysis (LY30). Blue lines represent the MA and LY30 values obtained for donor A, who did not show an increased LY30 upon incubation with EspP^WT^.

All donors with the exception of one (donor A) exhibited a time-dependent reduction in MA and increase in LY30 relative to BSA or buffer control when whole blood was treated with EspP^WT^ (Fig 5). In all donors, treatment of whole blood with EspP^S263A^ did not result in a significant change in MA or LY30 relative to BSA or buffer control (Fig 5).

Evaluation for LPS revealed significantly higher LPS levels in purified EspP^WT^ and EspP^S263A^ compared to PBS-G buffer and BSA controls, as would be expected from purification of an *E*. *coli* recombinant protein (S1 Fig). While PBS-G buffer and BSA had LPS levels of 0.024 and 0.032 U/mL (P = 0.396), respectively, EspP^WT^ and EspP^S263A^ had LPS levels of 1,092 and 839 U/mL (P<0.001 and P = 0.001, respectively, relative to PBS-G; P<0.001 and P = 0.001, respectively, relative to BSA). EspP^WT^ and EspP^S263A^ did not have significantly different LPS levels (P = 0.115).

## Discussion

In their initial characterization of this protease, Brunder *et al*. showed that EspP cleaved human coagulation FV and hypothesized that EspP may induce coagulopathy via this inactivation of FV. Our results indeed demonstrate a time- and concentration-dependent reduction in factor V activity by EspP^WT^, but not by EspP^S263A^, indicating that the active site of the proteolytic domain of EspP is responsible for the inactivation of factor V. In addition, EspP also cleaves coagulation factor VIII, which may further contribute to the coagulopathy. When we tested the effect of EspP on coagulation in whole blood and plasma, EspP^WT^, but not EspP^S263A^, significantly reduced the coagulation activities of factors V and VIII as expected but also significantly reduced the activities of factors VII and XII in a time- and concentration-dependent manner. In contrast, prothrombin activity, although significantly reduced, remained above 0.80 U/mL, which seems unlikely to be physiologically relevant. Unsurprisingly, we also observed substantial variability in the ability of EspP to reduce activities between coagulation factors. Reduction in activity appeared to be the most pronounced in factor VII, followed by FV, FVIII, and FXII, suggesting that EspP may act in a non-discriminatory way. Substitution of whole blood with plasma did not produce a significant difference in reduction of coagulation factor activities, suggesting that the cellular components of blood (i.e. platelets, erythrocytes, leukocytes) do not play a significant role in the reduction of coagulant activities by EspP^WT^. The sum of EspP’s effect on the coagulation factors is reflected in the time- and concentration-dependent increase of all three common measures of secondary hemostasis: PT, aPTT, and TT.

Incubation of whole blood with EspP^WT^ and EspP^S263A^, but not PBS-G or BSA controls, resulted in a time-dependent accelerated rate of platelet-fibrin clot formation, as suggested by the reduced reaction time (R-time) and clot formation time (K-time) and increased clot kinetics (α-angle). However, purified EspP^WT^ and EspP^S263A^, but not PBS-G or BSA, were found to have high levels of LPS contamination. Because LPS itself can accelerate the rate of platelet-fibrin clot formation [13,14], the accelerated rate of platelet-fibrin clot formation observed in whole blood incubated with EspP^WT^ and EspP^S263A^ could therefore be explained by contamination from LPS.

**Fig 6:**
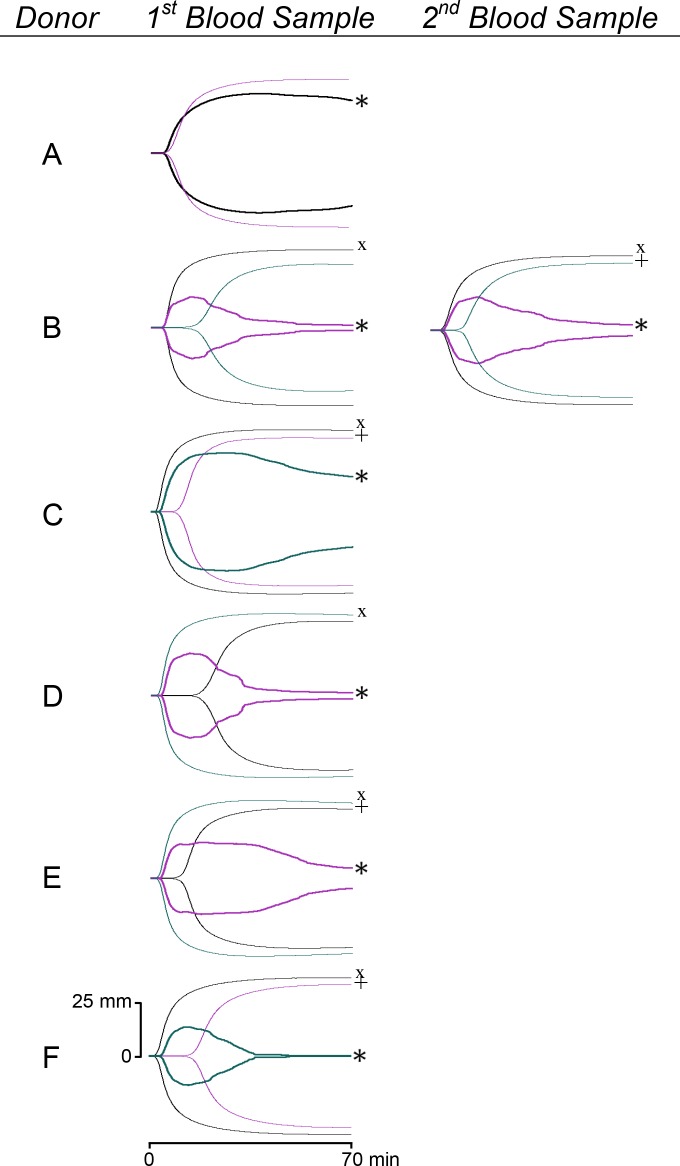
Thrombelastograph of whole blood from 6 donors after treatment with EspP^WT^, EspP^S263A^, or BSA. Shown are the thrombelastographs, monitored for a duration of 70 min following reconstitution with calcium chloride, of blood samples treated with EspP^WT^ (*), EspP^S263A^ (×), and BSA (+).

Incubation of whole blood with EspP^WT^, but not EspP^S263A^, BSA, or buffer control, resulted in an accelerated rate of fibrinolysis as evidenced by a decreased MA and increased LY30, suggesting that the active site of the protease may be responsible for this effect. MA is a measure of the maximum clot strength and is dependent on both the rate of clot formation and lysis, while LY30 is a measure of clot lysis 30 min after MA is reached, and provides a marker for fibrinolysis. Visual examination of the thrombelastograph tracings confirmed the presence of fibrinolysis (Fig 6). However, this effect was observed in only five of the six individuals tested. This suggests the tantalizing possibility that there may be a subset of individuals in the population who are resistant to the fibrinolysis-enhancing effects of EspP. Additional studies with larger sample sizes are required to address this hypothesis.

The time-dependent reduction in stability of human whole blood *in vitro* at a physiologically relevant temperature (37°C) led to the need to incubate samples with EspP^WT^ or EspP^S263A^ at concentrations between 0.1 and 1 mg/mL. This is not without precedent as previous experiments examining the effect of proteolysis in biological samples have employed similar concentrations of protease [15,16].

The observed enhanced fibrinolysis and reduced clot strength suggests a potential pathogenic role for EspP in D+HUS. Evidence for accelerated fibrinolysis in D+HUS has been equivocal. Van Geet *et al*. found an elevation in markers of fibrinolysis (t-plasminogen activator, u-plasminogen activator, and D-dimer) but no difference in PT, aPTT, factor VIII, and von Willebrand factor antigen level in an analysis of 24 D+HUS patients compared to 15 patients with acute renal failure as controls [17]. A number of studies [18–21] reported elevated levels of plasminogen-activator inhibitor type 1, an inhibitor of fibrinolysis, in patients with HUS. It may be that reduction in coagulation factor activities induced by EspP only occurs in an *in vitro* setting since such a phenomenon has not yet been reported in an *in vivo* setting. The current prevailing thought in the pathogenesis of hemorrhagic colitis is the induction of apoptosis of intestinal epithelial cells by Stx leading to the destruction of the gastrointestinal (GI) mucosal membrane [22,23]. Given that patients with EHEC infection have antibodies to EspP, suggesting direct contact between the protease and the bloodstream [3,24,25], one possible role for the coagulopathic effect of EspP may be to facilitate the invasion of Stx into the circulatory system by maintaining a coagulopathic and pro-fibrinolytic state at the site of damaged intestinal epithelium, and consequently the promotion of hemorrhagic colitis.

Other evidence that supports a pathogenic role for EspP in D+HUS includes cleavage of human apolipoprotein A-I [26], which acts as a stabilizing factor for prostacyclin (PGI_2_) [27], an inhibitor of platelet activation [28]; downregulation of complement activation by cleavage of C3/C3b and C5 which may facilitate adherence [29] and colonization of the EHEC in the gut by protecting the bacteria from opsonization and complement-mediated lysis [30]; and EspP forming rope-like fibers with cytopathic and adhesive properties [31]. Paradoxically, EspP has also been shown to cleave and inactivate EHEC haemolysin [32], a pore-forming cytolysin that damages microvascular endothelial cells and is thought to play a role in the pathogenesis of hemorrhagic colitis and D+HUS [32,33]. Thus the results from this study and others suggest that EspP may have a role in the pathogenesis of hemorrhagic colitis and D+HUS, but is neither a necessary nor sufficient virulence factor in the genesis of the disease. It is most likely that EspP may act through many different mechanisms and in concert with other virulence factors like Stx, (CDT)-V, EHEC-hly, and subtilase cytotoxin to enhance the virulence of EHEC in the formation of D+HUS.

The present study surveyed the effects of EspP on the coagulation system, platelet-fibrin clot formation and fibrinolysis. Further work is required to elucidate the proteolytic cleavage sites for EspP on the coagulation factors and whether the enhanced fibrinolysis observed following treatment with EspP is directly or indirectly caused by this protease. This will aid in better understanding the mechanisms by which EspP may influence the pathogenesis of D+HUS.

## Supporting Information

S1 Fig LPS levels in samples. PBS-G, BSA (0.1 mg/mL), EspP^WT^ (0.1 mg/mL), and EspP^S263A^ (0.1 mg/mL) were assayed for LPS contamination as described in Materials and Methods. (A) Visible light photograph of microtiter plate wells following incubation with Quanti-Blue. Shown are wells containing known concentrations of LPS (standards) as well as wells containing varying dilutions of PBS-G, BSA, EspP^WT^, and EspP^S263A^. (B) Standard curve obtained from the data shown in (A). (C) LPS concentrations obtained from three consecutive experiments, with individual data points indicated by closed dots, means indicated by the height of the gray bars, and standard errors of the means indicated by the error bars.
(TIF)
