# Supplementary figures and images for: EspP, an Extracellular Serine Protease from Enterohemorrhagic E. coli, Reduces Coagulation Factor Activities, Reduces Clot Strength, and Promotes Clot Lysis
Source: PLoS One. 2016 Mar 2;11(3):e0149830. doi: 10.1371/journal.pone.0149830 (PMC4775034; doi:10.1371/journal.pone.0149830)

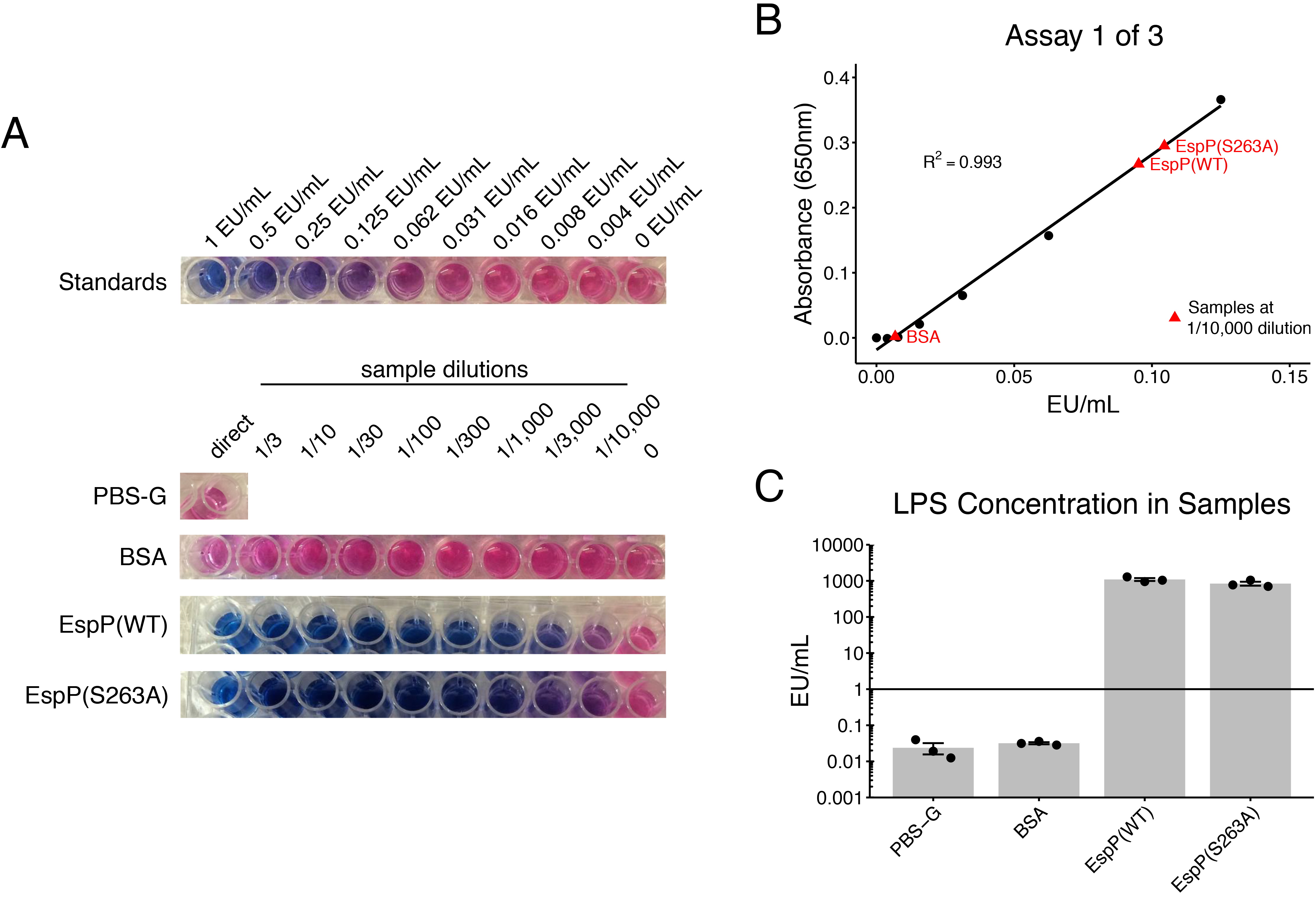

Supplement: S1 Fig — PBS-G, BSA (0.1 mg/mL), EspPWT (0.1 mg/mL), and EspPS263A (0.1 mg/mL) were assayed for LPS contamination as described in Materials and Methods. (A) Visible light photograph of microtiter plate wells following incubation with Quanti-Blue. Shown are wells containing known concentrations of LPS (standards) as well as wells containing varying dilutions of PBS-G, BSA, EspPWT, and EspPS263A. (B) Standard curve obtained from the data shown in (A). (C) LPS concentrations obtained from three consecutive experiments, with individual data points indicated by closed dots, means indicated by the height of the gray bars, and standard errors of the means indicated by the error bars. (TIF) [file pone.0149830.s001.tif]
